# Effect of Glomerular Filtration Rate by Different Equations on Prediction Models for End-Stage Renal Disease in Diabetes

**DOI:** 10.3389/fendo.2022.873318

**Published:** 2022-06-03

**Authors:** Liangjing Lv, Xiangjun Chen, Jinbo Hu, Jinshan Wu, Wenjin Luo, Yan Shen, Rui Lan, Xue Li, Yue Wang, Ting Luo, Shumin Yang, Qifu Li, Zhihong Wang

**Affiliations:** Department of Endocrinology, The First Affiliated Hospital of Chongqing Medical University, Chongqing, China

**Keywords:** estimated GFR, diabetic kidney disease, ESRD prediction model, revised Lund–Malmö equation, glomerular filtration rate

## Abstract

**Background and Objectives:**

The study aimed to evaluate the performance of a predictive model using the kidney failure risk equation (KFRE) for end-stage renal disease (ESRD) in diabetes and to investigate the impact of glomerular filtration rate (GFR) as estimated by different equations on the performance of the KFRE model in diabetes.

**Design, Setting, Participants, and Measurements:**

A total of 18,928 individuals with diabetes without ESRD history from the UK Biobank, a prospective cohort study initiated in 2006–2010, were included in this study. Modification of diet in renal disease (MDRD), chronic kidney disease epidemiology collaboration (CKD-EPI) or revised Lund–Malmö (r-LM) were used to estimate GFR in the KFRE model. Cox proportional risk regression was used to determine the correlation coefficients between each variable and ESRD risk in each model. Harrell’s C-index and net reclassification improvement (NRI) index were used to evaluate the differentiation of the models. Analysis was repeated in subgroups based on albuminuria and hemoglobin A1C (HbA1c) levels.

**Results:**

Overall, 132 of the 18,928 patients developed ESRD after a median follow-up of 12 years. The Harrell’s C-index based on GFR estimated by CKD-EPI, MDRD, and r-LM was 0.914 (95% CI = 0.8812–0.9459), 0.908 (95% CI = 0.8727–0.9423), and 0.917 (95% CI = 0.8837–0.9496), respectively. Subgroup analysis revealed that in diabetic patients with macroalbuminuria, the KFRE model based on GFR estimated by r-LM (KFRE-eGFR_r-LM_) had better differentiation compared to the KFRE model based on GFR estimated by CKD-EPI (KFRE-eGFR_CKD-EPI_) with a KFRE-eGFR_r-LM_ C-index of 0.846 (95% CI = 0.797–0.894, p = 0.025), while the KFRE model based on GFR estimated by MDRD (KFRE-eGFR_MDRD_) showed no significant difference compared to the KFRE-eGFR_CKD-EPI_ (KFRE-eGFR_MDRD_ C-index of 0.837, 95% CI = 0.785–0.889, p = 0.765). Subgroup analysis of poor glycemic control (HbA1c >8.5%) demonstrated the same trend. Compared to KFRE-eGFR_CKD-EPI_ (C-index = 0.925, 95% CI = 0.874–0.976), KFRE-eGFR_r-LM_ had a C-index of 0.935 (95% CI = 0.888–0.982, p = 0.071), and KFRE-eGFR_MDRD_ had a C-index of 0.925 (95% CI = 0.874–0.976, p = 0.498).

**Conclusions:**

In adults with diabetes, the r-LM equation performs better than the CKD-EPI and MDRD equations in the KFRE model for predicting ESRD, especially for those with macroalbuminuria and poor glycemic control (HbA1c >8.5%).

## Introduction

Diabetes mellitus (DM) is the leading cause of end-stage renal disease (ESRD) (1), and it is estimated that 20–40% of ESRD patients have DM. Approximately 30% of ESRD cases result from DM (2, 3). Diabetic patients with ESRD have a higher risk of death than patients with ESRD alone (4), and early identification effectively improves the prognosis (5). Therefore, the ability to predict ESRD in a population with diabetes is of great clinical importance.

Many studies have focused on ESRD prediction models (6–8), but only a few of them have focused on patients with DM. The existing ESRD prediction models that focus on patients with DM often require information for renal pathological characteristics, such as glomerular sclerosis score and tubular injury markers, or they contain complex variables, making it difficult to generalize for the clinical setting. There is a lack of simple and reliable predictive models for ESRD in diabetes. The kidney failure risk equation (KFRE) is a risk prediction model for ESRD widely used in the general population (9–11). The KFRE includes sex, age, urinary (or urine) albumin-to-creatinine ratio (UACR), and estimated glomerular filtration rate (eGFR) (9). The KFRE has also been recommended by the European Renal Best Practice (ERBP), especially for older people (12), but whether it effectively measures the risk of incident ESRD in people with diabetes is less certain.

The glomerular filtration rate (GFR) is an effective indicator of renal function. Because of the complexity and invasiveness of its gold standard measurement, eGFR is often used as a surrogate in clinical practice. The Modification of Diet in Renal Disease (MDRD) and Chronic Kidney Disease Epidemiology Collaboration (CKD-EPI) equations are the most commonly used equations based on serum creatinine, but they do not perform well in DM (13–15). In 2011, a new eGFR equation based on serum creatinine, named the revised Lund–Malmö (r-LM), emerged. Zafari et al. evaluated the diagnostic performance of GFR estimated by the CKD-EPI, MDRD, and r-LM equations in diabetes, and they found that the r-LM equation outperformed the CKD-EPI MDRD equations in terms of consistency, precision, accuracy, and bias in estimating GFR in adults with diabetes (16). Nevertheless, it remains unclear whether using different eGFR equations affects the performance of the ESRD prediction model and whether the GFR estimation formula is better in DM for predicting ESRD. Therefore, using clinical data from the UK Biobank prospective cohort, this study aimed to investigate the performance of GFR as estimated by different formulas in the KFRE model to predict the risk of ESRD in patients with diabetes.

## Methods

### Cohorts

The UK Biobank is a large-scale biomedical database containing genetic and health information from 502,536 participants aged 37 to 73 in the UK between 2006 and 2010 (17). Baseline clinical data, such as demographics, were collected *via* a touchscreen device (17). This cohort is supported by the North West Multi-Centre Research Ethics Committee (REC Reference: 11/NW/03820). All participants provided informed consent. The current analysis was approved by the UK Biobank (ID: 66536).

Patients with a prior history of ESRD (defined as the date of ESRD algorithm reporting earlier than the date of recruitment), patients whose eGFR was less than 15 ml/min/1.73 m^2^ and patients without a history of diabetes (defined as the date of onset of diabetes earlier than the date of recruitment) were excluded.

### Clinical Factors and Measurements

All clinical data were derived from the UK Biobank cohort. Blood and urine samples were collected and analyzed in the central laboratory. Blood glucose and glycated hemoglobin levels were measured by chromatographic analysis using the Variant II Turbo system (Bio-Rad, USA). Total cholesterol (TC), triglyceride (TG), high-density lipoprotein cholesterol (HDL-c), and low-density lipoprotein cholesterol (LDL-c) were measured by chemical analysis using an AU5800 instrument (Beckman Coulter, Brea, CA, USA). Serum creatinine (Scr) and urinary creatinine were measured using an AU5400 instrument (Beckman Coulter) (18). Urine microalbumin was measured by an immunoturbidimetric method using reagents and calibrators obtained from Randox Bioscience (19). Ethnicity data were sorted by black or other to calculate GFR estimated by the CKD-EPI (20) and MDRD equations (21) as shown in **Table 1**.

**Table 1:** The three models and variables.

Model	Variables	eGFR Equations	
Model 1	age, sex, UACR, eGFR_CKD-EPI_	Black	Female & SCr ≤62 µmol/L: 166×(Scr/62)^−0.329^ × 0.993^Age^
			Female & SCr >62 µmol/L: 166×(Scr/62) ^−1.209^ × 0.993^Age^
			Male & SCr ≤80 µmol/L: 163×(Scr/80) ^−0.411^ × 0.993^Age^
			Male & SCr >80 µmol/L: 163×(Scr/80) ^−1.209^ × 0.993^Age^
		Non-Black	Female & SCr ≤62 µmol/L: 144×(Scr/62) ^−0.329^ × 0.993^Age^
			Female & SCr >62 µmol/L: 144×(Scr/62) ^−1.209^ × 0.993^Age^
			Male & SCr ≤80 µmol/L: 141×(Scr/80) ^−0.411^ × 0.993^Age^
			Male & SCr >80 µmol/L: 141×(Scr/80) ^−1.209^ × 0.993^Age^
Model 2	age, sex, UACR, eGFR_MDRD_	Black	175×Cr ^-1.154^×Age ^-0.203^ (×0.742 if female) × 1.212
		Non-Black	175×Cr ^-1.154^×Age ^-0.203^ (×0.742 if female)
Model 3	age, sex, UACR, eGFR_r-LM_		eX^−^0.0158 × age + 0.438 × ln(age)
			Female & SCr <150 µmol/L: X = 2.50 + 0.0121 × (150^−^SCr)
			Female & SCr ≥150 µmol/L: X = 2.50 − 0.926 × ln(SCr/150)
			Male & SCr <180 µmol/L: X = 2.56 + 0.009 68 × (180^−^SCr)
			Male & SCr ≥180 µmol/L: X = 2.56 − 0.926 × ln(SCr/180)

Scr, serum creatinine. Scr unit is μmol/L.

### Definition

The ESRD outcome was defined using the ICD-10 and OPCS4 hospital admission codes. Participants who reached stage 5 CKD, required renal replacement therapy, or received peritoneal dialysis were identified as ESRD patients during the follow-up period. This algorithm has previously been used to successfully identify patients with ESRD in the UK Biobank (22).

No albuminuria, microalbuminuria, and macroalbuminuria was defined as UACR <30 mg/g, 30 mg/g< UACR <300 mg/g, and UACR >300 mg/g, respectively. Good, fair, and poor blood glucose control were defined as HbA1c <7%, 7%< HbA1c <8.5%, and HbA1c >8.5%, respectively.

### Statistical Analysis

This study discussed the following predictive models for ESRD in diabetes (**Table 1**). The descriptive statistics were grouped by gender. The Kolmogorov–Simov Z test was used to test the normality of continuous variables. All the continuous variables in this study were not normally distributed, and they are expressed as the median (interquartile range, IQR). Intergroup comparisons were performed using the t test or Mann–Whitney U test. Categorical variables are expressed as percentages (frequency), and the chi-square test was used for comparison between groups. The correlation between each variable and the outcome was tested by Spearman’s test. Cox proportional risk regression was used to observe the correlation coefficients between each variable and ESRD risk in each model. Harrell’s C-index and net reclassification improvement (NRI) index were used to evaluate the differentiation of the models. Subgroup analysis was performed on the basis of renal function and blood glucose levels. All the analyses were performed by Stata 15 software (Statacorp LP, College Station, TX, USA) and R v4.0.4 (http://www.R-project.org, The R Foundation). P <0.05 represents statistical significance.

## Results

### Cohort Description

**Figure 1:**
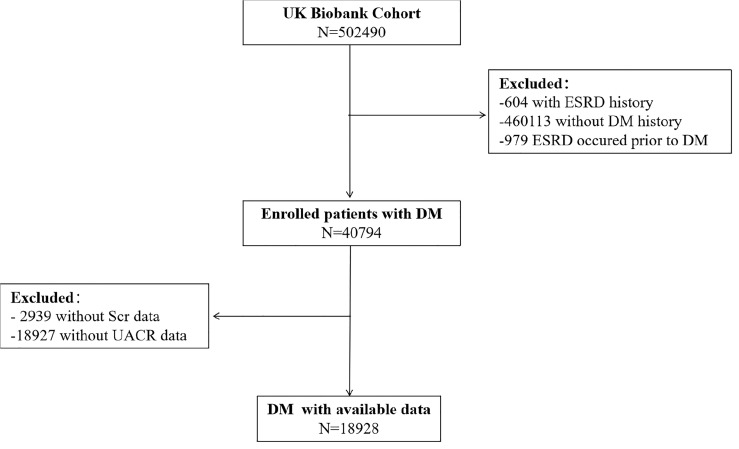
Flow chart of study population.

Of the half of a million people in the UK Biobank, 604 had a history of ESRD, 460,113 did not have a history of diabetes, and 40,794 were eligible for inclusion in this study. Among them, 2,939 lost serum creatinine data, 18,927 lost urine microalbumin data, and 18,928 had complete data and were included in this study (**Figure 1**). At baseline, the age of the male and female participants was 60 (53, 67) and 59 (52, 67) years old, respectively. The HbA1c of the male and female participants was 46.81 (28.47, 65.15) mmol/mol and 47.81 (29.96, 65.66) mmol/mol, respectively. The other baseline characteristics are summarized in **Table 2**.

**Table 2:** Baseline characteristics, follow-up and outcome of the UK Biobank cohort with diabetes.

Characteristics		Female (n = 6,597)	Male (n = 12,331)	P-value
Age (years)		59 (52, 67)	60 (53, 67)	0.000
Ethnicity	White	5,606 (85.0)	10,871 (88.2)	0.000
	Black	49 (0.7)	62 (0.5)	
	Asian	343 (5.2)	702 (5.7)	
	Other	599 (9.1)	696 (5.6)	
Smoking status (%, smoker)		333 (5.0)	12,331 (100.0)	0.000
BMI (kg/m^2^)		33.16 (26.56, 39.76)	31.66 (26.4, 36.92)	0.000
SBP (mmHg)		145 (125, 165)	147 (127, 166)	0.000
DBP (mmHg)		83 (72, 94)	84 (73, 95)	0.000
HbA1c (mmol/mol)		46.81 (28.47, 65.15)	47.81 (29.96, 65.66)	0.000
HDL-c (mmol/L)		1.18 (0.7, 1.66)	1.02 (0.61, 1.43)	0.000
LDL-c (mmol/L)		3.21 (2.25, 4.17)	2.93(2.03, 3.83)	0.000
TG (mmol/L)		2.25(1.03, 3.47)	2.44 (1.01, 3.87)	0.000
TC (mmol/L)		5.22 (3.96, 6.48)	4.75 (3.54, 5.96)	0.000
Scr (nmol/L)		65.72 (47.39, 84.05)	82.93 (59.56, 106.3)	0.000
UACR	<30	5,084 (77.1)	9,198 (74,6)	0.000
	30–300	1,352 (20.5)	2,704 (21.9)	
	>300	161 (2.4)	429 (3.5)	
eGFR (ml/min/1.73 m^2^)	CKD-EPI	88.64 (71.44, 105.84)	88.16 (71.24, 105.08)	0.067
	MDRD	86.85 (64.54, 109.16)	88.42 (66.3, 110.54)	0.000
	r-LM	80.8 (65.26, 96.34)	78.88 (63.74, 94.02)	0.000
Insulin (%, Yes)	Yes	814 (12.3)	1,590 (12.9)	0.274
Statin (%, Yes)	Yes	3,627 (55.0)	7,690 (62.4)	0.000
ESRD outcome	0	6,559 (99.4)	12,237 (99.2)	0.142
	1	38 (0.6)	94 (0.8)	

Quantitative variables are shown as median (interquartile range), and qualitative parameters are presented as numbers with the percentage in parentheses.

BMI, body mass index; SBP, systolic blood pressure; DBP, diastolic blood pressure; FPG, fasting plasma glucose; HbA1c, glycated hemoglobin; HDL-c, high-density lipoprotein cholesterol; LDL-c, low-density lipoprotein cholesterol; TG, total triglyceride; TC, total cholesterol; Scr, serum creatinine; UACR, urinary albumin creatinine ratio; eGFR, estimated glomerular filtration rate.

Most of the participants had normal renal function at baseline, but the GFR estimated by different formulas varied. After a median follow-up of 12 years, 132 of 18,928 patients developed ESRD during the follow-up period.

### Association of eGFR Based on Different Equations With ESRD Outcomes

Each eGFR was negatively correlated with the outcome of ESRD (**Supplementary Table 1**). Regardless of the formula, a decreased eGFR was associated with an increased risk of ESRD (**Table 3**). After the Z conversion, the increased risk of ESRD due to decreased eGFR still existed (**Table 3**).

**Table 3:** Adjusted hazard ratio of variables in different models for ESRD.

	N	HR (10 ml/min/1.73 m^2^)	95% CI	HR (1 SD)	95% CI
KFRE	18,928				
eGFR_CKD-EPI_		0.916	0.909–0.924	0.227	0.198–0.259
eGFR_MDRD_		0.913	0.905–0.921	0.132	0.109–0.159
eGFR_r-LM_		0.906	0.899–0.914	0.223	0.195–0.253

Cox proportional hazard models adjusted for age, sex, UACR and eGFR by different equations.

In diabetes, compared to patients with well-controlled blood glucose, those who had poor blood glucose control were associated with a greater risk of ESRD in response to the decrease in the eGFR (**Supplementary Table 2**).

### Prediction of ESRD in Diabetes by Different Models

The C-index of the original KFRE model (age, sex, UACR, and eGFR_CKD-EPI_) was 0.914 (0.8812–0.9459). The eGFR estimated by the r-LM equation improved the differentiation of the ESRD prediction model (C-index of 0.917 (0.8837–0.9496), P = 0.165; **Table 4**). The NRI index was calculated, but the results showed that the eGFR calculated by different formulas did not significantly impact the differentiation of the KFRE for predicting ESRD (**Supplementary Table 3**).

**Table 4:** Harrell’s C-index of the three models for ESRD.

Model	Participants	Cases	C-index	95% CI	P-value
KFRE	18,928	132			
eGFR_CKD-EPI_			0.914	(0.8812–0.9459)	NA
eGFR_MDRD_			0.908	(0.8727–0.9423)	0.977
eGFR_r-LM_			0.917	(0.8837–0.9496)	0.165

C-index for Cox proportional hazard models adjusted for age, sex, UACR and eGFR by different equations.

NA, not applicable.

### Subgroup Analysis: Diabetes With Different Kidney Conditions

According to the UACR values, the data were further divided into three groups as follows: no albuminuria, microalbuminuria, and macroalbuminuria. Compared with the no albuminuria and macroalbuminuria subgroups, the original KFRE model based on eGFR_CKD-EPI_ performed best for predicting ESRD in diabetic patients with microalbuminuria [C-index of 0.923 (0.873–0.973); **Table 5**].

**Table 5:** Harrell’s C-index of the different models for ESRD according to UACR subgroups.

Model	Albuminuria					
	No albuminuria		Microalbuminuria		Macroalbuminuria	
	C-index	P	C-index	P	C-index	P
KFRE						
eGFR_CKD-EPI_	0.839 (0.736–0.943)		0.923 (0.873–0.973)		0.839 (0.788–0.890)	
eGFR_MDRD_	0.829 (0.716–0.942)	0.735	0.922 (0.871–0.973)	0.565	0.837 (0.785–0.889)	0.765
eGFR_r-LM_	0.831 (0.722–0.941)	0.724	0.923 (0.872–0.974)	0.494	0.846 (0.797–0.894)	0.025

C-index for Cox proportional hazard models adjusted for age, sex, UACR and eGFR by different equations.

Regarding the impact of different eGFR equations, there was no significant difference in the C-index of the KFRE model for predicting ESRD in the no albuminuria and microalbuminuria groups. However, in the macroalbuminuria subgroup, the differentiation of the model based on eGFR_r-LM_ was better than the other formulas [C-index of 0.846 (0.798–0.859), P = 0.025; **Table 5**].

### Subgroup Analysis: Diabetes With Different Glycemic Control Levels

According to the HbA1c values, the data were further divided into three groups as follows: good blood glucose control, fair blood glucose control, and poor blood glucose control. Comparison among groups showed that the KFRE model performed best for predicting ESRD in diabetic patients with fair blood glucose control, as indicated by an HbA1c range of 7–8.5% [C-index of 0.954 (0.925–0.984); **Table 6**].

**Table 6:** Harrell’s C-index of different models for ESRD according to HbA1c subgroups.

Model	HbA1c (mmol/mol)					
	<53.0		53.0–69.39		>69.39	
	C-index	P-value	C-index	P-value	C-index	P-value
KFRE						
eGFR_CKD-EPI_	0.901 (0.849–0.954)		0.954 (0.925–0.984)		0.925 (0.874–0.976)	
eGFR_MDRD_	0.898 (0.843–0.953)	0.697	0.946 (0.909–0.984)	0.925	0.925 (0.876–0.974)	0.498
eGFR_r-LM_	0.901 (0.847–0.955)	0.543	0.955 (0.924–0.985)	0.449	0.935 (0.888–0.982)	0.071

C-index for Cox proportional hazard models adjusted for age, sex, UACR and eGFR by different equations.

Regarding the impact of different eGFR equations, there was no significant difference in the C-index of the KFRE model for predicting ESRD in the good and fair blood glucose control groups. In the poor blood glucose control subgroup, however, the differentiation of the model based on eGFR_r-LM_ was better than the other formulas [C-index of 0.935 (0.888–0.982), P = 0.071; **Table 6**].

## Discussion

In this study, UK Biobank data were used to evaluate the predictive performance of the KFRE model for ESRD in diabetes and to investigate the impact of different eGFR equations. We found that the KFRE effectively identified patients with a high risk of ESRD (C-index of 0.8812–0.9459). Overall, the GFR formulas did not significantly impact the performance of the KFRE in our data. However, subgroup analysis showed that in diabetic patients with macroalbuminuria (UACR >300 mg/g) and poor blood glucose control (HbA1c >8.5%), the prediction model based on eGFR_r-LM_ outperformed the prediction models based on eGFR_CKD-EPI_ and eGFR_MDRD_ in identifying diabetic patients with a higher risk of ESRD [C-index of 0.846 (0.797–0.894) and 0.935 (0.888–0.982), respectively].

Previous studies have developed and applied many ESRD prediction models, but only a few studies have focused on diabetes, a serious public health problem. Tangri et al. (9) observed the occurrence of renal failure in patients with stage 3–5 CKD (approximately 40% with diabetes); they showed that a model adjusted for serum albumin, serum calcium, blood phosphate, and blood bicarbonate well predicts the incidence of ESRD (C-index of 0.917, 95% CI (0.901–0.933)), but this model is difficult to promote in clinical practice because it uses variables not commonly used in daily follow-up. Jardine et al. (23) enrolled patients with type 2 diabetes from the ADVANCE study (n = 11,140) and performed a 5-year follow-up to observe the risk of renal-related outcomes (creatinine doubling and onset proteinuria); their model contains ten variables (gender, race, eGFR, UACR, SBP, antihypertensive therapy, HbA1c, diabetic retinopathy, waist circumference, and education age) that effectively identifies patients with higher risk of ESRD (C-index of 0.815–0.880), but some of the variables, such as diabetic retinopathy and education age, require a long-term follow-up, limiting its clinical application. Another study has developed a model, a so-called renal risk score, to predict ESRD in patients with diabetes (n = 25,736); this model includes gender, race, age, duration of diabetes, proteinuria, serum creatinine, systolic blood pressure, HbA1c, smoking status, and previous history of cardiovascular disease with a C-index of 0.89–0.92 for ESRD (24), but it also contains many variables that limit its clinical application. In contrast, the KFRE not only performed well in identifying high-risk patients with diabetes but also used more convenient and easily obtained variables.

All the mentioned studies used the CKD-EPI formula to estimate GFR. Although this formula is widely used, its accuracy is poor in patients with diabetes, particularly in the subgroup with poor renal function (14). Therefore, this study also investigated the impact of different eGFR formulas on the performance of the prediction model. Our results showed that the prediction model based on eGFR_r-LM_ better identified diabetic patients with a high-risk for ESRD. Although the GFR formulas did not significantly impact the performance of the KFRE in this study, it may have been due to the uneven distribution of this population. At baseline, most participants had normal or fair renal function, which may have underestimated the proportion of patients with kidney disease. Additionally, diabetic patients with macroalbuminuria (UACR > 300 mg/g) and poor blood glucose control (HbA1c >8.5%) had a higher risk of incident ESRD (4). Subgroup analysis revealed that the prediction models based on eGFR_r-LM_ outperformed the prediction model based on eGFR_CKD-EPI_ and eGFR_MDRD_ in identifying patients who are at high-risk for ESRD, indicating that r-LM is a better option for estimating the GFR in a prediction model for ESRD in diabetes.

This study had the following advantages: This was the first study to investigate the impact of different GFR formulas on a prediction model. Additionally, the study population was obtained from the UK Biobank cohort, representing a large sample size and high reliability of the results. Moreover, the recommended model is simple, and the required variables (age, sex, UACR, and eGFR_r-LM_) can be easily obtained in routine clinical follow-ups.

Nevertheless, this study had several limitations. First, most of the study population had normal renal function (**Table 1**), which does not reflect the continuous spectrum of renal function in diabetes. Additionally, the population was mainly white, indicating that the use of this model for other races, such as the Asian population, requires further validation. Second, we defined diabetes mellitus by the first occurrence data of the UK Biobank database, indicating that the diabetic subtypes were not distinguished. However, this model may be suitable to some extent for all types of diabetes.

## Conclusion

In this study, the KFRE was validated for predicting ESRD in diabetes. We found that estimating the GFR using different equations had little impact on the model performance. However, eGFR_r-LM_ performed better in subgroups with macroalbuminuria and poor glycemic control. These findings may provide theoretical support for the early identification and intervention of ESRD in diabetes.

## Data Availability Statement

The original contributions presented in the study are included in the article/**Supplementary Material**. Further inquiries can be directed to the corresponding author.

## Ethics Statement

The studies involving human participants were reviewed and approved by the North West Multi-Centre Research Ethics Committee. The patients/participants provided their written informed consent to participate in this study.

## Author Contributions

ZW proposed the conception,QL and SY helped with the study design. LT and YW take part in the execution, acquisition of data. XL, RL, YS and WL analysis and interpretation the data, JW, JH and XC took part in drafting, revising or critically reviewing the article; LL take charge of data analysing and drafting and revising articles. All authors gave final approval of the version to be published.

## Funding

This work was supported by the Technological Innovation and Application Development Project of Chongqing (cstc2019jscx-msxmX0207), the Chongqing Science and Health Joint Medical Research Project (2020FYYX141; 2018GDRC004), the Innovative Funded Project of Chongqing Innovation and Retention Program (cx2019032),the Chinese Foundation for International Medical Exchange(Z-2017-26-1902-2) and the Chongqing Yong and Middle-aged Senior Medical Talents Studio (ZQNYXGDRCGZS2021001).

## Conflict of Interest

The authors declare that the research was conducted in the absence of any commercial or financial relationships that could be construed as a potential conflict of interest.

## Publisher’s Note

All claims expressed in this article are solely those of the authors and do not necessarily represent those of their affiliated organizations, or those of the publisher, the editors and the reviewers. Any product that may be evaluated in this article, or claim that may be made by its manufacturer, is not guaranteed or endorsed by the publisher.
