# Supplementary material for: Effect of Glomerular Filtration Rate by Different Equations on Prediction Models for End-Stage Renal Disease in Diabetes
Source: Front Endocrinol (Lausanne). 2022 Jun 3;13:873318. doi: 10.3389/fendo.2022.873318 (PMC9205244; doi:10.3389/fendo.2022.873318)
Supplement: Supplementary file 1 [file DataSheet_1.pdf]

Supplement Table 1 Spearman's correlation coefficient analysis of variables and ESRD endpoint

	r	P
age	0.008	0.261
sex	0.011	0.142
uacr	0.1118	0.000
ckdepi	-0.112	0.000
mdrd	-0.112	0.000
fas	-0.113	0.000
rlm	-0.113	0.000

Supplement Table 2 Adjusted hazard ratio of varients in different models for ESRD by different HbA1c level

Model	HbA1c(mmol/mol)） <53.0 （<7%）		53.0-69.39 （7%-8.5%）		>69.39 (>8.5%)	
	HR	95%CI	HR	95%CI	HR	95%CI
KFRE						
$eGFR_{CKD\text{-}EPI}$	0.908	0.898-0.919	0.916	0.909-0.924	0.926	0.912-0.941
$eGFR_{MDRD}$	0.905	0.893-0.916	0.913	0.905-0.921	0.932	0.918-0.946
$eGFR_{r\text{-}LM}$	0.898	0.887-0.909	0.906	0.899-0.914	0.917	0.901-0.932

Cox proportional hazard models adjusted for age sex uacr and eGFR by different equation

Supplement Table 3 Net reclassification improvement of different models for ESRD

Model	Case NRI		Noncase NRI
KFRE	$eGFR_{CKD\text{-}EPI}$	NA	NA
	$eGFR_{MDRD}$	0.000000(-0.499921,0.000000)	0.000000(0.000000,0.000106)
	$eGFR_{r\text{-}LM}$	0.000000(0.000000,0.000000)	0.000000(-0.000053,0.000053)
